# Reemergence of Dengue Virus Type 4, French Antilles and French Guiana, 2004–2005

**DOI:** 10.3201/eid1211.060339

**Published:** 2006-11

**Authors:** Philippe Dussart, Anne Lavergne, Gisèle Lagathu, Vincent Lacoste, Jenny Martial, Jacques Morvan, Raymond Cesaire

**Affiliations:** * Institut Pasteur de la Guyane, Cayenne, French Guiana;; † Centre Hospitalier Universitaire de Fort de France, Fort de France, Martinique, French West Indies

**Keywords:** Dengue-4, phylogeny, French Antilles, French Guiana, dispatch

## Abstract

After 10 years of absence, dengue virus type 4 (DENV-4) has recently reemerged in Martinique, Guadeloupe, and French Guiana. Phylogenetic analyses of strains isolated from 2004 to 2005 showed that they belong to DENV-4 genotype II, but to a different cluster than strains isolated from 1993 to 1995.

Dengue is a viral disease transmitted by mosquitoes. It is caused by any of the 4 viral serotypes of dengue virus (DENV), designated DENV-1, DENV-2, DENV-3, and DENV-4. DENV belongs to the genus Flavivirus and family Flaviviridae. Flaviviruses are enveloped, single-stranded, positive-sense RNA viruses. The genomic RNA is ≈11 kb and has 10 genes coding 3 structural proteins (capsid [C], envelope [E], and membrane [M]), and 7 nonstructural proteins (NS1, NS2a, NS2b, NS3, NS4a, NS4b, and NS5).

Dengue is the predominant arthropodborne viral disease affecting humans. DENV causes a wide range of symptoms from inapparent or mild disease (dengue fever) to severe forms such as dengue hemorrhagic fever (DHF) and dengue shock syndrome (DSS). The disease is now endemic in >100 countries and threatens >2.5 billion people. It currently occurs in tropical areas and affects <100 million persons each year (*1*). Of these persons, 500,000 have DHF and ≈25,000, mainly children, die. The World Health Organization has estimated a 30-fold incidence increase in dengue the past 50 years (*2*).

DENV is transmitted to humans by Stegomyia aegypti (formerly Aedes aegypti) mosquitoes. Before the 1980s, epidemic dengue was rare in the Americas because St. aegypti had been eradicated from most Central and South American countries. In the 1990s, St. aegypti had almost completely reinvaded the regions in which it was found before its eradication. Within the past 30 years, the increase in the worldwide transportation network, as well as uncontrolled population growth and urbanization, has led to larger and more frequent DENV epidemics and more cases of DHF/DSS (*3*).

During the past 20 years, the 4 DENV serotypes have been isolated in the French departments of the Americas: Guadeloupe, Martinique, and French Guiana. Martinique and Guadeloupe are 2 Caribbean islands located in the Lesser Antilles and represent the French West Indies, and French Guiana is located southeast of the French West Indies in northern South America between Suriname and Brazil. DENV is endemic in French Guiana. Dengue epidemics occurred in this country at 4–6-year intervals from the 1960s to the early 1990s (*4*). The first DHF cases were associated with a DENV-2 outbreak in 1991–1992 (*5*). DENV-4 was sporadically isolated between 1993 and 1995. Cocirculation of DENV-1 and DENV-2 caused outbreaks between 1996 and 1998, followed by 2 consecutive DENV-3 epidemics in 2001–2002 and 2004–2005. Martinique had 3 dengue epidemics during the past decade in a setting of sporadic transmission and seasonal peaks from July to December. These epidemics were associated with DENV-2 and DENV-4 in 1995, DENV-1 in 1997, and DENV-3 in 2001 (*6*). The epidemiology of DENV in Guadeloupe is similar to that in Martinique, although no data are available about previous dengue epidemics.

## The Study

Our study reports the evolution of DENV-4 in French Guiana and the French Caribbean islands since its last detection and circulation from 1993 to 1995. We studied 8 DENV-4 strains isolated from human sera from French Guiana in 1993 and 1995 (n = 6) and in 2004 and 2005 (n = 2) (Table). We also tested 3 human serum specimens from Martinique (n = 2) and Guadeloupe (n = 1) that were positive for DENV-4 during dengue surveillance in the fourth quarter of 2004.

**Table:** Dengue virus type 4 strains used for the phylogenetic reconstruction

Country of origin	Strain	Year of isolation	GenBank accession no.
French Guiana	FGU-Dec-1993	1993	DQ390322
	FGU-Mar-1994, -Jun-1994, Sep-1994	1994	DQ390325, DQ390327, DQ390329
	FGU-Feb-1995, -Jun-1995	1995	DQ390324, DQ390326
	FGU-Oct-2004	2004	DQ390328
	FGU-Fev-2005	2005	DQ390323
Guadeloupe	GUA-FWI-Dec-2004	2004	DQ390320
Martinique	MAR-FWI-Aug-2004, Dec-2004	2004	DQ390319, DQ390321
Bahamas	BAH 1998 A, B, C	1998	AY152364–66
Barbados	BDS 1993 A, B	1993	AY152375–76
	BDS 1999	1999	AY152368
Costa Rica	CRA 1996	1996	AY152104
Dominica	DOM 1981	1981	AY152360
Ecuador	ECD 1994	1994	AY152292
El Salvador	ELS 1993	1993	AY152300
Honduras	HON 1991	1991	AY152379
Jamaica	JAM 1981	1981	AY152389
	JAM 1983	1983	AY152384
Martinique	MAR-FWI 1995	1995	AY152100
Mexico	MEX 1991	1991	AY152378
	MEX 1995	1995	AY152304
Monserrat	MON 1994 A, B, C, D	1994	AY152369–71
Puerto Rico	PR 1982 M03, M05	1982	AY152336, AY152344
	PR 1985 M32, M33	1985	AY152856–57
	PR 1986 115	1986	AY152224
	PR 1987 67, 73	1987	AY152236, AY152268
	PR 1990 96	1990	AY152855
	PR 1992 24, 35, 36	1992	AY152112, AY152188, AY152208
	PR 1994 81, 83	1994	AY152144, AY15148
	PR 1998 13, 17	1998	AY152056, AY152068
Suriname	SUR 1982 A, B, C, D	1982	AY152385–88
	SUR 1994 A, B, C	1994	AY152372–74
Trinidad	TRI 1982 A, B	1982	AY152382–83
	TRI 1984 A, B	1984	AY152380–81
	TRI 1994	1994	AY152377
	TRI 1999	1999	AY152367
Venezuela	VEN 1995	1995	AY152092

DENV-4 infection was confirmed by using virus isolation on AP 61 cells as previously described (*7*). We then amplified from first-passage RNA a 1,940-bp region of the genome for the E gene and adjacent prM/M and NS1 junctions previously described (*8*) to analyze the phylogenetic relationships of these strains. Each PCR product was cloned by using the TOPO TA Cloning kit (Invitrogen, Paisley, UK). For each isolate, 3 clones were sequenced by Genome Express (Meylan, France).

We compared our 11 sequences to the 87 DENV-4 sequences available in the GenBank database. Sequences were aligned by using MacVector version 7.2 software (Oxford Molecular Ltd., Oxford, UK). Percentage of nucleotide identity between sequences was determined for all observed differences (including insertions and deletions) and was calculated for the 1,473 nt available for all 98 sequences analyzed. Sequence comparison of strains from Martinique, Guadeloupe, and French Guiana with those reported from Asia showed that nucleotide divergence is 8%–12%. The level of nucleotide divergence was lower for sequences from the Caribbean area (maximum nucleotide divergence 2.8%). All 11 newly obtained sequences were different from each other. These sequences exhibited a 0.06%–0.47% nucleotide divergence (strains isolated in 1993 and 1995) and a 0.06%–1.22% nucleotide divergence (strains isolated in 2004 and 2005) among themselves. These strains had amino acid changes at position 351 in the E gene (from isoleucine to valine) and position 52 in the NS1 gene (from lysine to arginine), which supports their inclusion in the modern Caribbean basin clade (*8*).

We conducted phylogenetic analyses on nucleotide sequences by using distance and parsimony methods in PAUP* version 4.0b8 to increase the reliability of derived tree topologies (*9*). We also used the neighbor-joining distance matrix algorithm with the Kimura 2 parameter (Figure) and the heuristic algorithm for the maximum parsimony analysis (data not shown). Robustness of resulting topologies was examined by using bootstrap analyses (*10*). Both neighbor-joining and parsimony algorithms underwent 1,000 iterations.

**Figure:**
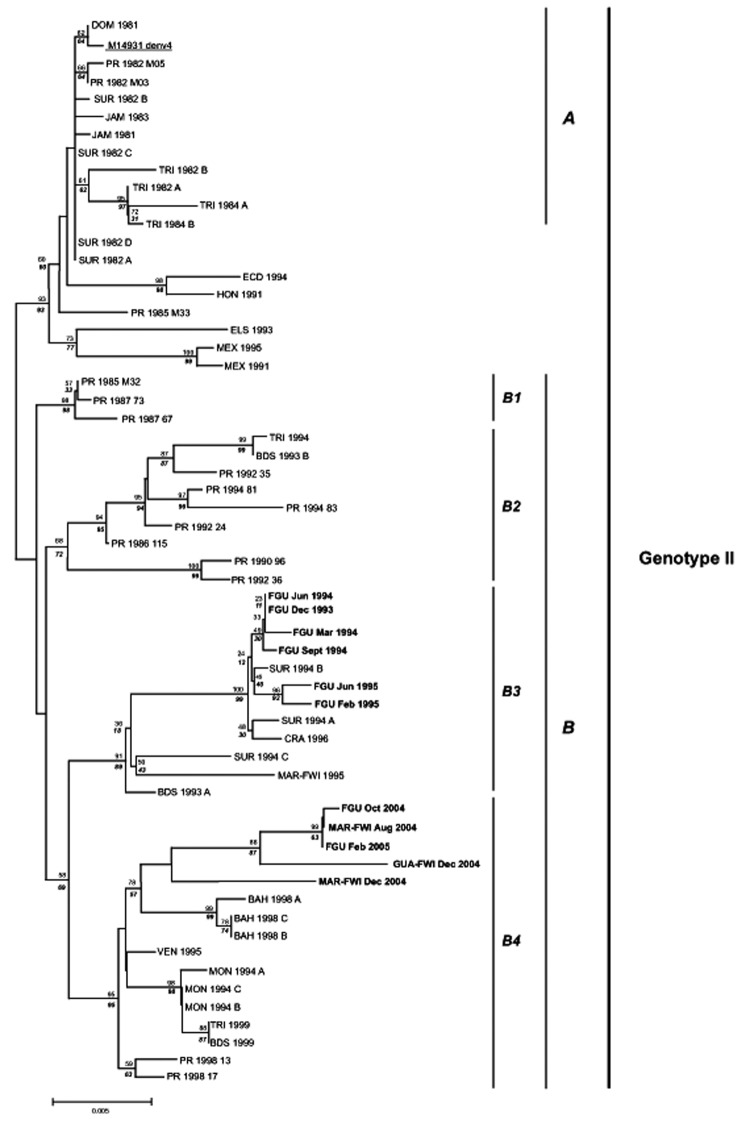
Phylogenetic tree of American dengue virus type 4 (DENV-4) strains generated by using the neighbor-joining algorithm with Kimura 2 parameter distance. Numbers above branches refer to bootstrap values generated by using distance, and numbers under nodes refer to bootstrap values generated by using parsimony. Names of isolates refer to country and are listed in the Table. Accession no. M14931 refers to the complete genome of DENV-4. A and B refer to the 1981 introduction group and to the modern Caribbean clade, respectively, and B1–B4 correspond to 4 different lineages within group B, as previously reported by Foster et al. (*8*).

The phylogenetic tree constructed with the neighbor-joining method on nucleotide sequences restricted to all American DENV-4 isolates available in GenBank (Table) is shown in the Figure. Our 11 strains segregate into 2 main clades that were identified in the 2 phylogenetic analyses from consistent topologic associations and high bootstrap values. The strains isolated from French Guiana in 1993 and 1994 clustered with strains from Suriname and Costa Rica, which were isolated at the same time, as well as with a previously reported strain from Martinique isolated in 1995. Our 5 strains isolated in 2004 and 2005 are related to a group of DENV-4 genotype II sequences isolated in the Bahamas in 1998. This finding suggests that strains circulating in French departments of the Americas in 2004 and 2005 probably evolved from strains detected in the Caribbean islands during the late 1990s. Our observations confirm that DENV-4 lineages in the Caribbean and nearby regions are grouped temporally rather than by the geographic origin of the isolates, as has been previously suggested (*8*).

## Conclusions

The DEN-4 serotype was first reported in the Americas in 1981. At that time, isolates collected in the Americas were similar to strains collected in Southeast Asia. This lineage was designated DENV-4 genotype II (*11*). Since it was first detected in Dominica and the French islands of Saint Barthelemy and Saint Martin (*12*), this genotype has spread rapidly throughout the Caribbean and Latin America and caused DF and sporadic cases of DHF/DSS (*3*).

DENV-4 genotype II never stopped circulating in the Caribbean region, despite its being periodically locally eliminated (*8*). This genotype reappeared in French departments of the Americas in 2004 after the introduction of a strain circulating in neighboring Caribbean islands, rather than by reemergence of a strain detected 10 years ago. Furthermore, this DENV-4 genotype was detected in French Guiana (October 2004) a few weeks after it was detected in Martinique (August 2004), which suggests that the strain came from the Antilles (Table). A similar phenomenon had already been observed in 1999 for the DENV-3 genotype (J. Morvan, unpub. data). This model of viral spread may be caused by cultural and economic ties between French departments, which allows gene flow through the Caribbean islands and South America as persons with viremia move through the region. This reflects the general pattern of dengue evolution in the Americas (*13**,**14*).

Reintroduction of DENV-4 in the French Antilles and French Guiana in 2004 after a decade of absence highlights the risk for subsequent epidemics in persons with no immunity to dengue. As expected, the French Antilles had a major DENV-4 epidemic with cocirculation of DENV-2 from July 2005 to January 2006 (*15*). In contrast, only sporadic cases of DENV-4 infection have been observed in French Guiana since the beginning of 2005. This indicates that other factors such as mosquito density or human susceptibility, in addition to immune status of the host or silent transmission of the virus, can modulate the risk for dengue epidemics.
